# Future Landscape of Anti-Claudin 18.2 Antibodies in Gastric Adenocarcinoma

**DOI:** 10.3390/antib14010026

**Published:** 2025-03-18

**Authors:** Wendy M. Covert, Jane E. Rogers

**Affiliations:** Department of Pharmacy Clinical Programs, The University of Texas MD Anderson Cancer Center, Houston, TX 77030, USA; jerogers@mdanderson.org

**Keywords:** claudin 18.2, zolbetuximab, gastric adenocarcinoma

## Abstract

Advanced gastric adenocarcinoma (GAC) carries a poor prognosis. Targeted therapy in GAC has traditionally been limited to anti-human epidermal growth factor receptor-2 and anti-vascular endothelial growth factor agents. Recent years have brought immune checkpoint therapy to the GAC treatment landscape. However, continued discovery of targeted therapy in GAC is needed. Claudins, transmembrane proteins located in tight junctions of epithelial and endothelial cells, help regulate cellular polarity. Claudin dysregulation has been linked to cancers and other diseases. Claudin 18.2 specifically has become a new novel and exciting biomarker for GAC. Many agents are in the investigative pipeline, including monoclonal antibodies, antibody-drug conjugates, bispecific antibodies, and chimeric T-cell therapy. Recently, zolbetuximab, an anti-claudin 18.2 monoclonal antibody, was the first of these agents to get FDA approval. Here, we review zolbetuximab’s place in therapy along with other agents being explored.

## 1. Introduction

Gastric adenocarcinoma (GAC) remains a health concern as it is the fifth most frequently diagnosed cancer and the third leading cause of cancer-related deaths worldwide [1]. The incidence varies depending on geographic location, with the highest incidence rates in Asia, South and Central America, and Eastern Europe. GAC continues to be seen in Western countries, but due to a lack of cancer screening modalities and standards, GAC is frequently diagnosed in the advanced setting in these areas. GAC patients diagnosed with metastatic spread carry a <10% 5-year relative survival [2], and therefore, advancements in treatment are necessary.

Biomarker driven therapy has improved outcomes and become the standard of care practice in metastatic GAC [1]. All patients diagnosed should undergo microsatellite instability (MSI) testing by PCR/next-generation sequencing (NGS) or mismatch repair by immunohistochemistry (IHC). Patients that are MSI-High (MSI-H) or deficient in MMR (dMMR) will receive immune checkpoint inhibitor therapy [1]. MSI-H/dMMR metastatic GAC has an incidence of <5% [3], however, MSI-H/dMMR GAC patients will have a different course of management compared to MSI-stable/proficient MMR (pMMR) GAC patients [1]. Metastatic GAC patients will additionally be tested for human epidermal growth factor receptor-2 (HER2) overexpression using IHC or fluorescence in situ hybridization (FISH) [1]. Patients who are HER2 positive (~15% of GAC patients) will have the option of adding HER2-directed therapy added to a traditional chemotherapy combination of platinum plus fluoropyrimidine [1,4]. HER2-positive patients are also treated differently than HER2-negative patients due to additional treatment options like other HER2-directed therapies and clinical trials [4]. Lastly, all patients should have programmed-death ligand-1 (PD-L1) testing [1]. PD-L1 testing has varied among front-line metastatic GAC studies [5,6,7], but an accepted analysis utilizes a combined positive score (CPS). CPS is determined by the number of PD-L1 stained cells (tumor cells, lymphocytes, macrophages) divided by the total number of viable tumor cells multiplied by 100 [1]. Patients with PD-L1 CPS positive tumors will have options for immune checkpoint therapy to be added to traditional platinum plus fluoropyrimidine.

As of October 2024, metastatic GAC had zolbetuximab, an anti-claudin 18.2 monoclonal antibody, approved for addition to front-line platinum plus fluoropyrimidine for patients who are claudin-18.2 (CLDN18.2) positive [8]. Now, all patients should obtain CLDN18.2 expression, given this option. With current drug target approval, HER2-negative tumors that test positive for CLDN18.2 are candidates for zolbetuximab. CLDN18.2 positivity is defined as patients with ≥75% of tumor cells with moderate to strong membranous CLDN18 staining [8,9]. CLDN18.2 expression in the advanced GAC setting is reported at ~40% [9].

Before the discovery of CLDN18.2, targeted therapy in advanced GAC has been limited to HER2-directed therapy, trastuzumab and trastuzumab deruxtecan, and anti-vascular endothelial growth factor receptor-2, ramucirumab [1]. Additionally, CLDN18.2 has shown to be a novel biomarker target for GAC. The upcoming pipeline for this target is overflowing with additional monoclonal antibodies (mAb), antibody-drug conjugates (ADCs), bispecific antibodies (BsAbs), and chimeric antigen receptor T-cell (CAR-T) therapy [10]. Navigating these upcoming developments will be the key to success.

## 2. Targeting Claudin 18.2 in GAC

### 2.1. Claudin 18.2 Role in Normal Gastric Mucosa

Epithelia, sheets of cells that line organ surfaces and body structures, including the skin, gastrointestinal tract, and respiratory tract, act as physical and chemical barriers within the body [11,12]. Endothelial cells function similarly to epithelial cells but are found at the interface between vascular and perivascular compartments, such as blood vessels and lymphatic vessels. These cells are connected through a complex of intercellular junctions. At the apical cell interfaces, the complex junctions form the *zonula occludens*, also known as the tight junctions. The *zonula adhaerens* lies in the intermediary junction between cells, and the *macula adhaerens* forms the basal cell surface complexes. Tight junctions (TJ) are paracellular connections between contiguous cells that form a diffusion barrier to maintain cellular polarity [13]. Within the TJ are transmembrane proteins called occluding, tricellulin, and various claudins (CLDNs).

CLDNs, first identified in 1998 by Tsukita et al., are a family of transmembrane proteins located in the tight junctions of epithelial and endothelial cells [11]. CLDNs help regulate cellular polarity and act as paracellular barriers affecting tight junction permeability in the intercellular space. CLDN18.2, one of two isoforms of CLDN 18, expression is restricted to the tight junctions of healthy gastric mucosa and not exposed on cell surfaces; however, malignant transformation of healthy gastric mucosa can lead to ectopic expression [14]. Thus, agents targeting CLDN18.2 would theoretically have limited off-target effects due to the surface inaccessibility of CLDN18.2 in healthy cells. Surface expression and dysregulation of CLDN18.2 have been reported in GAC, esophageal, pancreatic, and lung cancers, making CLDN18.2 a potentially viable therapeutic target for these cancers [15,16]. CLDN18.2 expression has been measured using IHC assays, but the definition of clinically relevant positivity varies amongst clinical trials [17,18,19,20,21]. CLDN18.2 prevalence in GAC and gastroesophageal (GEAC) cancers ranges from 24–44% positivity, defined as ≥75% of tumor cells staining moderate-to-strong via IHC testing [9,22,23,24,25]. Given CLDN18.2’s expression specifically in gastric mucosa, this makes for a new research hotspot in GAC. In comparison to HER2, the incidence of CLDN18.2 expression is higher, making it an area of direct focus for GAC. Additionally, when compared to PD-L1, CLDN18.2 higher expression has shown at least with the monoclonal antibody, zolbetuximab, to be a direct correlation to more efficacy as there remains controversy in PD-L1 scoring and method regarding immune checkpoint therapy impact. The incidence and exposure of CLDN18.2 in GAC make for an exciting target in GAC.

### 2.2. Current Anti-Claudin 18.2 Antibody Experience in GAC

Currently, there are a plethora of trials seeking to evaluate the safety and efficacy of various CLDN18.2 biotherapeutics in GEAC patients [26]. Here, we review the current progress.

#### 2.2.1. Monoclonal Antibodies

##### Zolbetuximab

Zolbetuximab, formerly IMAB362 or claudiximab, is a first-in-class chimeric IgG_1_ mAb that binds CLDN18.2 leading to apoptosis via antibody-dependent cellular cytotoxicity (ADCC) and complement-dependent cytotoxicity (CDC) [27]. First described in the phase II MONO trial, zolbetuximab showed modest efficacy as a single agent in the treatment of patients with CLDN18.2-positive (2+/3+ staining in ≥50% of tumor cells) advanced GEACs [17]. The overall response rate (ORR) was 9%. In the moderate-to-high CLDN18.2 expression subgroup (≥70% of tumor cells), 14% of patients achieved a partial response (PR) and 17% had stable disease (SD) with zolbetuximab treatment. Following the MONO trial, the FAST trial evaluated the use of first-line epirubicin, oxaliplatin, and capecitabine (EOX) with or without zolbetuximab for patients with CLDN18.2-positive (2+/3+ IHC in ≥40% of tumor cells) advanced GEAC [19]. Significant improvement in progression-free survival (PFS) and overall survival (OS) occurred in the zolbetuximab group (median PFS 7.5 months vs 5.3 months *p* < 0.0005); median OS 13 months vs 8.3 months *p* < 0.0005). Subgroup analysis of patients with moderate-to-strong CLDN18.2 expression (≥70% of tumor cells) showed a clear improvement in PFS and OS in the zolbetuximab plus EOX arm versus EOX alone, whereas patients with 40–69% moderate to strong CLDN18.2 positivity had no significant difference in outcomes. Because patients with moderate-to-strong expression (≥70% of tumor cells) were the driving force behind zolbetuximab’s beneficial results, future zolbetuximab clinical trials would focus investigative efforts in this population. The FAST trial gave knowledge that with CLDN18.2 mAb targeting, the main benefit to continue exploring is in patients with high CLDN 18.2 expression.

The multi-cohort phase II ILUSTRO trial [18] was conducted to determine if the addition of zolbetuximab to immunotherapy or standard chemotherapy was beneficial. Patients with high (2+/3+ ICH ≥ 75% of tumor cells) or intermediate (2+/3+ IHC ≥ 50% but <75% of tumor cells) CLDN18.2 expression advanced or GAC or gastroesophageal junction adenocarcinoma (GEJ) were divided into one of three cohorts: (1) high CLDN18.2 expression patients for third-line or later zolbetuximab monotherapy, (2) high CLDN18.2 expression, HER2-negative patients for first line zolbetuximab plus FOLFOX, or (3) high or intermediate CLDN18.2 expression patients for third-line or later zolbetuximab plus pembrolizumab. All patients in Cohorts 1 and 2 had high CLDN18.2 expression. The ORR for Cohort 1 was 0%, and the disease control rate (DCR) was 44.4% (95% CI 25.48–64.67) via independent review. Median PFS was 1.54 months in this heavily pretreated population. Patients with prior gastrectomy and diffuse-type disease by Lauren classification had longer OS of 6.64 months and 9.89 months, respectively. Cohort 2 patients had an ORR of 71.4% and a DCR of 100% by independent review. The median PFS was 17.8 months. Unfortunately, Cohort 3, which only had 3 patients, reported no response to treatment. No new adverse effects were reported for these cohorts. An additional cohort has been announced as part of an expansion of the ILUSTRO trial. Cohort 4 will evaluate the combination of first-line zolbetuximab, mFOLFOX6, and nivolumab in intermediate-to-high CLDN18.2 positivity (2+/3+ IHC in ≥75% of tumor cells [high] or ≥50% but <75% of tumor cells [intermediate]), HER2-negative metastatic GAC/GEJ patients.

After success in phase II trials, zolbetuximab was studied versus placebo in combination with chemotherapy as first-line treatment in two phase III trials, SPOTLIGHT and GLOW [20,21]. Both studies defined CLDN18.2 positivity as ≥75% staining in tumor cells. In the SPOTLIGHT trial, zolbetuximab or placebo was given with fluorouracil, oxaliplatin and folinic acid [levofolinate in Japan] (FOLFOX) to patients with CLDN18.2 positive, HER-2 negative advanced/metastatic GAC/GEJ [20]. Zolbetuximab treatment resulted in a statistically significant prolongation of median PFS of 10.61 months versus 8.67 months, *p* = 0.0066. Additionally, there was a significant increase in median OS of 18.23 months versus 15.54, *p* = 0.0053. In the GLOW trial, patients with CLDN18.2 positive, HER-2 negative advanced/metastatic GAC/GEJ received zolbetuximab or placebo with capecitabine and oxaliplatin (CAPOX) [21]. Median PFS for the zolbetuximab group was statistically significant at 8.21 months versus 6.80 months, *p* = 0.0007, and median OS was also statistically significantly prolonged in patients receiving zolbetuximab (median OS 14.39 months vs. 12.16 months, *p* = 0.0118). Treatment-related adverse events (TRAEs) reported in both the SPOTLIGHT and GLOW trials were like the adverse events seen in earlier trials, with nausea and vomiting occurring more frequently in the zolbetuximab arms [17,19,20,21]. SPOTLIGHT and GLOW provided the conclusion that zolbetuximab has benefit to front-line chemotherapy in high expressed CLDN18.2 patients. These trials also gave insight into zolbetuximab’s role as a potential maintenance therapy for patients that have disease control on the chemotherapy combination. As oxaliplatin has a well-known maximum of doses that can be given due to cumulative neuropathy, maintenance therapy is an important area of investigation. These trial results led to the approval of zolbetuximab for the treatment of adult patients with HER-2 negative advanced/metastatic GAC/GEJ whose tumors are CLDN18.2 positive in Japan, the United Kingdom, the European Union, and the United States [28]. Table 1 summarizes the trials associated with zolbetuximab use in mG/GEJ patients.

Significant nausea with and without vomiting reported with zolbetuximab administration is thought to be due to direct effects on the gastric tissue. A prior study in ferrets revealed on-target, off-tumor effects on the gastric mucosal surface after zolbetuximab [29]. Fosaprepitant, a neurokinin-1 (NK-1) receptor antagonist, was found to be effective in reducing emesis and reduced damage to gastric tissue when given in combination with other antiemetic agents. Zolbetuximab was typically administered over 2 h in reported trials, with patients experiencing significant nausea and vomiting during infusions [17,18,19,20,21]. The onset of nausea and/or vomiting typically occurred within the first 60 min of the initial zolbetuximab infusion [30]. Patients with prior gastrectomy reported less nausea and vomiting than those without prior gastrectomy [20,21]. Reported nausea and vomiting decreased with further exposure to zolbetuximab. Commercial labeling of zolbetuximab recommends infusions be administered over 3.5–4.5 h [31]. Although recommended premedications should consist of a combination of antiemetic agents, which may include NK-1 receptor antagonists and 5-HT3 receptor antagonists, clearer guidance on the management of nausea and vomiting from zolbetuximab has been recently published [32]. Premedication regimens should include an NK-1 antagonist, 5-HT3 antagonist, and dexamethasone with or without olanzapine. An alternative regimen of a 5-HT3 antagonist, dexamethasone, and olanzapine may also be considered. Slowing or pausing infusions is recommended for any patients experiencing nausea with or without vomiting during zolbetuximab infusions. With these robust antiemetic recommendations and the potential for extended infusion times when given in combination with other therapies, we wonder if reduced emetic potential will be a deciding factor on which other anti-CLDN18.2 inhibitors have future commercial success.

##### Osemitamab

Formerly known as TST001, osemitamab is a second-generation humanized IgG_1_ anti-CLDN18.2 mAb currently under investigation for metastatic GAC/GEJ cancer [10,33,34,35,36,37]. Osemitamab exerts action via ADCC and CDC leading to cell death in a similar fashion as zolbetuximab but has an optimized Fc segment design that produces a stronger affinity to bind tumor cells [33]. Stronger affinity may lead to improved ADCC and CDC effects, specifically in tumors with low to moderate CLDN18.2 expression. The phase I/IIa TranStar101 trial evaluated the safety and tolerability of osemitamab as monotherapy (Part A) or in combination with nivolumab and/or FOLFOX (Part B) for a cohort of patients with advanced/metastatic GAC/GEJ [33]. Recently updated data showed the most common TRAEs overall were nausea (73.5%), vomiting (48.5%), and fatigue (30.9%), and grade ≥ 3 or greater TRAEs occurring in ≥3% of patients were nausea, vomiting, hypoalbuminemia, hypertension, infusion-related reaction, and increased lymphocyte count. The authors concluded that osemitamab monotherapy or in combination with nivolumab and/or FOLFOX was well tolerated.

TranStar102 is another multi-cohort phase I/II study being conducted to evaluate the efficacy and safety of osemitamab plus nivolumab or nivolumab plus CAPOX as first-line treatment for patients with advanced/metastatic GAC/GEJ [34,35,36,37,38]. Cohort C is evaluating osemitamab plus CAPOX as first-line therapy for CLDN18.2-positive (IHC ≥ 1+ in ≥10% of tumor cells), HER2-negative or unknown, advanced/metastatic GAC/GEJ [34,35]. PR was achieved in 28 of 42 evaluable patients. ORR between CLDN18.2 expression groups were similar. At the time of publication, 26 of 64 patients had progressive disease or death with a median PFS of 9.5 months. PFS stratified by CLDN18.2 expression was not calculable due to immature data at the time of reporting. In this cohort, there was no clear trend between PFS and CLDN18.2 expression levels. TRAEs included nausea (65.4%), hypoalbuminemia (65.4%), and vomiting (42.6%) with most reported as grade 1 or 2. Cohort G in the TranStar002 trial is evaluating first-line osemitamab in combination with nivolumab and CAPOX for patients with HER-2 negative or unknown, unresectable advanced/metastatic GAC/GEJ regardless of CLDN18.2 or PD-L1 expression [36,37]. Of the 66 patients with known PD-L1 and CLDN18.2 expression status, 37 patients had progressive disease or death with an ORR of 68%, 61.1%, and 50% in the moderate/high (M/H), low (L), and unknown/negative groups (U/N), respectively. Patients with M/H CLDN18.2 expression had a median PFS of 12.6 months compared to 8.5 months and 6.7 months in the L and U/N groups, respectively. There was a clear trend between CLDN18.2 expression and anti-tumor efficacy for this cohort. When comparing patients with M/H CLDN18.2 expression to patients with either expression U/N, the hazard ratio was reported to be 0.443 (95% CI 0.205–0.958). TRAEs were mostly grade 1 or 2 and like the adverse effects reported in Cohort C, such as nausea, vomiting, and hypoalbuminemia. A phase III trial evaluating the efficacy of first-line osemitamab versus placebo in combination with FOLFOX or CAPOX and nivolumab in patients with CLDN18.2-positive, HER2-negative, advanced/metastatic GAC/GEJ is being developed [38].

#### FG-M108

FG-M108, or M108, is a second generation anti-CLDN18.2 monoclonal antibody with enhanced target affinity and ADCC action. It was studied with CAPOX as a first-line treatment for CLDN18.2-positive (IHC1/2/3+ in ≥10% of tumor cells), HER2-negative, advanced/metastatic GAC/GEJ in a phase I/II trial [39,40]. As of April 2024, the reported ORR is 77.8% and DCR is 97.2% in patients with medium-to-high CLDN18.2 positivity (IHC 2+3+ in ≥40% of tumor cells). The median PFS was 9.6 months. In patients with low CLDN18.2 expression (IHC 1+/2+/3+ 10% and 2+/3+ < 40% of tumor cells), the median PFS was 5 months. TRAEs were reported as mostly grade 1 or 2, with decreased neutrophil count (76.9%), anemia (73.1%), decreased white blood cell (WBC) count (67.3%), decreased platelet count (63.5%), and hypoalbuminemia (59.6%) most reported. Based on these encouraging results, a phase III trial of FG-M108 plus CAPOX versus CAPOX as first-line treatment of medium/high CLDN18.2 expressing advanced/metastatic GAC/GEJ is underway [41].

##### ASKB589

ASKB589, a humanized IgG_1_ anti-CLDN18.2, was first studied in a phase I/II trial as monotherapy for pre-treated patients with medium-to-high CLDN18.2-positive advanced solid tumors (Part A) or in combination with CAPOX as first-line treatment for patients with medium-to-high CLDN18.2-positive mG/GEJ adenocarcinoma (Part B) [42,43]. There were 9 evaluable patients with advanced/metastatic GAC and GEJ in Part A. ORR was 22%, and DCR was reported at 89% for this subgroup of patients. In Part B, ORR was 75%, and DCR was 100%. The majority of TRAEs reported for ASK589 in Part A and B were grade 1 or 2 and were like other anti-CLDN18.2 mAbs. An additional phase I/II trial was conducted using ASKB589 in combination with CAPOX and sintilimab (anti-PD-1 mAb) for first-line treatment of CLDN18.2-positive advanced/metastatic GAC/GEJ. ORR was reported as 80%, and DCR was 100%. A phase III trial is underway to evaluate ASKB589 or placebo in combination with tislelizumab and CAPOX as front-line therapy in patients with CLDN18.2-positive advanced, recurrent, or mG/GEJ cancer [44].

Zolbetuximab represents the first agent in this class approved for use in the front-line setting in combination with chemotherapy for tumors expressing ≥75% CLDN18.2 expression. Ongoing trials with osemitamab, FG-M108, and ASKB589 may provide benefit for patients with <75% CLDN18.2 in combination with chemotherapy and immunotherapy. With similar adverse effect profiles among these compounds and no mAb comparator arms in current phase III trials, cost, administration logistics, and tolerability may be the deciding factors on which agent will be preferred in the clinical setting.

### 2.3. Additional Therapeutic Modalities for CLDN18.2-Positive GAC

Other agents such as BsAb, ADCs, and CAR-T have also been considered as viable therapeutic modalities to target CLDN18.2 [42]. BsAbs are antibodies that have an additional binding target to synergize targeted cytotoxicity beyond that of a conventional mAb [45]. Some anti-CLDN18.2 BsAbs use T-cell costimulatory molecules, like 4-1BB or CD3, to activate T-cell receptor (TCR) complexes and drive T-cell cytotoxicity once bound to CLDN18.2 [46]. ADCs consist of antibodies engineered to target a cancer cell surface antigen, like CLDN18.2, a linker, and a cytotoxic payload, typically an antimitotic agent like monomethyl auristatin E (MMAE) or a topoisomerase inhibitor such as SN-38 [47]. Rare but serious TRAEs, such as interstitial lung disease and hepatotoxicity, have been previously reported with ADCs due to on-target, off-tumor effects from the cytotoxic payloads [48]. A delicate balance must be formed between the efficacy and toxicity of these agents. CAR-T cells are bioengineered T-cells that express a chimeric antigen receptor (CAR) to allow these modified T-cells to identify and combat cancer cells independently of major histocompatibility complex engagement [49]. In autologous products, T-cells are apheresed from the patient, altered with the CAR, and proliferated in vitro. Patients then undergo lymphodepletion, typically with fludarabine and cytarabine, prior to receiving the autologous CAR-T cell infusion. While usually reversible, cytokine release syndrome (CRS) and immune effector cell-associated neurotoxicity syndrome (ICANS) are significant toxicities often requiring inpatient monitoring and supportive care, which can lead to substantial overall costs for these treatments [50,51]. Although success has been seen in hematologic malignancies, CAR-T cell therapy has yet to achieve effective disease control in patients with solid tumors [51]. After preclinical success, CLDN18.2 was deemed a suitable target to explore for CAR-T cell products in solid tumor patients [52]. In the race to discover new targeted therapies for CLDN18.2-positive cancer patients, these compounds may offer increased affinity and specificity for their intended targets, but additional toxicities may make commercial success more difficult to achieve.

#### 2.3.1. Bispecific Antibodies

##### Givastomig

Givastomig, also known as TJ033721, ABL111, or TJ-CD4B, is a BsAb that binds CLDN18.2 and activates 4-1BB, a costimulatory receptor on activated T cells and natural killer cells [53]. By restricting activation of 4-1BB to cells with CLDN18.2 positivity, the hope was to achieve targeted anti-tumor activity with a decreased potential for hepatotoxicity, which had previously occurred in patients treated with urelumab and utomilumab, 4-1BB agnostic mAbs [54,55]. Patients with metastatic GEAC were enrolled in the phase I study of givastomig in the treatment of patients with solid tumors, regardless of CLDN18.2 expression, to explore the safety and potential efficacy of givastomig [56]. The most recent data presented show an ORR of 16.3% and a DCR of 48.8% in heavily pretreated patients receiving givastomig monotherapy. Patients responding to treatment had CLDN18.2 expression ranging from 11–100%. Preliminary median PFS was 3 months. The most common TRAEs reported were nausea (25.6%), anemia (23.3%), and decreased WBC count (23.3%). Grade 3 transaminitis was reported in four patients, and grade 4 thrombocytopenia occurred in one patient. Phase Ib of the trial is currently being expanded to evaluate the efficacy of givastomig in combination with chemotherapy and nivolumab as first-line therapy for patients with HER2-negative, CLDN18.2-positive metastatic GEAC, with results expected to be reported in late 2025 [57].

##### IBI389

In addition to utilizing 4-1BB as a costimulatory target, other BsAbs called bispecific T-cell engager (BiTE) antibodies link antigens on tumor cells to CD3 on T-cells, which activates T-cell receptor (TCR) complexes and initiates apoptosis [58,59,60]. IBI389 is a CLDN18.2/CD3 BiTE antibody being evaluated in pretreated advanced solid tumors in a phase I dose escalation and expansion study. Preliminary efficacy in metastatic GAC/GEJ cancer patients with CLDN18.2 expression ≥10% (2+/3+ IHC) showed an ORR of 40% and DCR of 73.1%. Adverse effects with IBI389 were more severe than CLDN18.2/4-1BB BsABs, including grade ≥3 TRAEs in over half of all reported patients. The most reported TRAEs were increased gamma-glutamyl transferase (21.9%), decreased lymphocyte count (13.2%), and nausea (4.4%). Grade 3 CRS occurred in 57% of patients treated with IBI389. CRS, and potentially neurotoxicity, occurs from the T-cell engaging mechanism of BiTE antibodies, which leads to an uncontrolled inflammatory response from the body during and after treatment. If not managed aggressively and promptly, life-threatening toxicity may result [60]. Overall, IBI389 had a manageable safety profile, according to the investigators, despite the incidence of CRS, and promising efficacy in patients with CLDN18.2-positive metastatic GAC/GEJ.

#### 2.3.2. Other Solid Tumor Bispecific Antibodies

##### PM1032

Another CLDN18.2/4-1BB BiTE compound currently under investigation in a phase I trial for the treatment of pretreated advanced or metastatic solid tumors, regardless of CLDN18.2 expression, is PM1032 [61,62]. In patients with evaluable CLDN18.2 expression, 66.7% of trial patients were positive. The ORR was 20% in CLDN18.2-positive metastatic GAC/GEJ. Available safety data stated the most common TRAEs were nausea (20%) and aspartate transaminase increase (16.7%). Further development of PM1032 is currently in progress.

##### QLS31905

A phase I trial was conducted to define the safety and efficacy of QLS31905, a CLDN18.2/CD3 BiTE antibody, in advanced solid tumors regardless of CLDN18.2 status [63]. ORR was 11.1%, and DCR was 63%, including nine gastric patients with stable disease. Fever (57.69%), nausea (50.00%), and decreased WBC count (34.62%) were the most common TRAEs. Grade 3 or higher TRAEs were reported in 40.38% of patients, and two patients had ≥grade 3 CRS. Although both IBI389 and QLS31905 had manageable toxicities and encouraging efficacy, the additional increased incidence of CRS and other grade 3 adverse effects will require close monitoring and supportive care. A phase I/II trial is being formed to evaluate QLS31905 plus chemotherapy as first-line treatment of patients with CLDN18.2-positive solid tumors [64].

##### Q1802

Currently, Q1802 is the only CLDN18.2/PD-L1 BsAb with preliminary data published for patients with pretreated advanced or metastatic solid tumors [65,66]. Since immunotherapy in combination with chemotherapy has become a backbone of treatment in mG/GEJ cancer, this compound may have great potential as a future therapy [1]. Of the nine gastrointestinal patients in the dose-expansion portion of the study, two achieved a partial response, and four achieved stable disease. TRAEs from Q1802 were mostly grade 1 or 2 and consisted of gastrointestinal AEs (89.7%), including nausea (62.1%), vomiting (62.1%), abdominal pain (27.6%), and gastroesophageal reflux disease (24.1%). Grade 3 TRAEs were reported in 24.1% of patients, with the most common grade 3 TRAEs being nausea and vomiting. Immune-related TRAEs were also reported but with a similar incidence to other commercially available immunotherapy agents.

The addition of costimulatory targets with BsAbs increases the potential for adverse effects like hepatotoxicity and CRS compared to single-targeted mAbs; however, dual targeting may increase the potential for treatment response in a patient population with a great need for additional therapeutic options. Because research is still in the preliminary stages, what place these agents will have in the treatment of GEAC remains to be seen. Additional anti-CLDN18.2 mAbs currently under investigation for the treatment of CLDN18.2-positive GEAC are included in Table 2.

#### 2.3.3. Antibody-Drug Conjugates

##### AZD0901

AZD0901, also known as CMG901, is an anti-CLDN18.2 ADC that exerts activity via monomethyl auristatin E (MMAE)-mediated cytotoxicity with bystander killing in addition to ADCC and CDC action [87,88,89]. In the phase I KYM901 trial, AZD0901 was studied in patients with advanced solid tumors, including metastatic GAC/GEJ cancer [87]. CLDN18.2 positivity was defined as IHC2+ in ≥5% tumor cells. In the dose-expansion portion of the study, 107 patients with GAC/GEJ cancer were treated with AZD901 monotherapy, with 73% of patients having prior exposure to immunotherapy and 5% receiving prior anti-CLDN18.2 treatment. Including metastatic GAC/GEJ patients from the dose escalation cohort, 113 patients were included in the response analysis of AZD901. Confirmed objective response rate (CORR) was 28%, and the DCR was 63%, with a median response duration of 7.9 months. Median PFS was reportedly 3.8 months, and median OS was 10.1 months at a median follow-up of 9 months. CLDN18.2 positivity of IHC 2+ in ≥20% of tumor cells was present in 97% of patients with CORR, which led researchers to make this cutoff the definition of CLDN18.2-high tumors. The combined CORR for GAC/GEJ patients in both dose escalation and expansion cohorts with CLDN18.2-high tumors was 33% compared to 5% for patients with CLDN18.2 positivity IHC 2+ in 5-<20% tumor cells. For CLDN18.2-high tumors, median PFS improved to 4.8 months and median OS improved to 11.8 months. Expansion cohort patients developed anemia (71%), hypoalbuminemia (61%), weight loss (59%), nausea (57%), vomiting (56%), decreased neutrophil count (53%), decreased WBC (51%), and decreased appetite (50%) as the most common TRAEs, and the most frequent grade ≥3 TRAEs were decreased neutrophil count (21%), anemia (14%), and vomiting (10%). The median onset time for nausea was 1 day and for vomiting was 2 days. Nausea lasted a median duration of 4 days, but both nausea and vomiting incidences declined with subsequent treatment cycles. Rates of nausea and vomiting were similar between patients with prior gastrectomy compared to patients without prior gastrectomy. Six types of prophylactic medications were administered to patients receiving AZD901. Grade 1–2 peripheral neuropathy occurred in 20% of patients, a known toxicity from MMAE. Overall, AZD901 showed promise as an additional anti-CLDN18.2 treatment option.

The follow-up phase II CLARITY-PanTumor01 trial is evaluating AZD0901 in patients with CLDN18.2-positive advanced solid tumors in multiple cohorts [88]. Cohort 1 will include HER2-negative metastatic GAC/GEJ patients with ≤2 lines of prior therapy. Cohort 2 will include previously untreated metastatic pancreatic cancer patients and will combine chemotherapy with AZD0901 treatment. In addition to the CLARITY-PanTumor01 trial, a phase III study is also currently under development titled CLARITY-Gastric01 [89]. This trial will evaluate the use of AZD0901 monotherapy vs. investigator choice chemotherapy for previously treated metastatic GAC/GEJ patients, including patients with prior exposure to CLDN18.2 mAb therapy.

##### LM-302

LM-302, or TPX-4589, is an ADC targeting CLDN18.2-positive cells using the same cytotoxic payload (MMAE) as AZD0901 with similar activity [90,91,92,93,94,95]. In a phase I/II trial, the safety and efficacy of LM-302 were evaluated in patients with pretreated advanced or metastatic GAC/GEJ cancer. Patients had to be CLDN18.2 positive, defined as IHC 2+/3+ in ≥50% of tumor cells, and to have been treated with two or more prior therapies. The ORR was 30.6% and DCR was 75%. The median PFS was 7.16 months. OS rate at 6 months was 95%. Common TRAEs reported for LM-302 were decreased WBC count (51.9%), decreased neutrophil count (51.1%), anemia (38.5%), vomiting (36.3%), and nausea (34.1%), with the most frequent grade ≥3 TRAEs reported as decreased neutrophil count (22.2%) and decreased WBC count (17.8%). LM-302 is currently being investigated in multiple phase II trials in combination with other anti-cancer therapies [92,93,94]. A phase III trial comparing LM-302 to the treating physician’s choice of chemotherapy (apatinib or irinotecan) for CLDN18.2-positive patients with metastatic GAC/GEJ cancer who have progressed on two or more lines of systemic therapy has also been activated [95].

#### 2.3.4. Other Solid Tumor Antibody-Drug Conjugates

##### EO-3021

EO-3021, or SYSA1801, is a CLDN18.2/MMAE ADC evaluated in a phase I trial for patients with CLDN18.2-positive, pretreated advanced solid tumors [96]. Overall ORR was 38.1%, and the DCR was 57.1%. In gastric cancer patients, the ORR was 47.1%, and the DCR was 64.7%. Any grade of TRAEs occurred in 75.8% of patients, and 24.2% were grade 3 or greater. TRAEs most commonly reported were nausea (42.4%), vomiting (36.4%), dry eye syndrome (21.2%), and anemia (21.2%). Two patients experienced grade 3 nausea and vomiting, which was labeled as a dose-limiting toxicity for EO-3021. Due to the anti-tumor activity reported in this phase I trial along with the acceptable toxicity profile, an additional phase I study is being performed outside of China to determine the optimal dose of EO-3021 in a more diverse patient population and the efficacy of it in mG/GEJ patients through an expansion cohort [97].

##### IBI343

IBI343 is an anti-CLDN18.2 ADC with an exatecan (topoisomerase I inhibitor) payload currently being studied in a phase I trial for patients with CLDN18.2-positive advanced or metastatic solid tumors [98,99]. Patients with CLDN18.2-positive (1+/2+/3+ IHC in ≥1% of tumor cells) mG/GEJ cancer were divided into a subgroup of the study. Overall ORR was 32.3%, and DCR was 75.8%. ORR was 37.5% and 44.8% in patients with moderate-to-high CLDN18.2 expression (2+/3+ IHC in ≥40% of tumor cells) at two different dose levels. DCR was 89.6% and 82.8%, and median PFS was 5.6 months and 5.5 months in these dosing groups. Of patients with high CLDN18.2 expression (2+/3+ in 75% of tumor cells), ORR was 46.7% and 52.9%, and DCR was 93.3% and 88.2% at the same two dose levels. Median PFS in the two dose levels explored for high CLDN18.2 expression patients was 6.8 months and 5.5 months. TRAEs from IBI343 occurred in 93.7% of patients, including grade 3 or higher TRAEs in 49.7% of patients. Anemia (54.7%), decreased WBC count (47.8%), and decreased neutrophil count (46.4%) were the most reported TRAEs, and grade 3 or higher gastrointestinal toxicities were rarely observed (vomiting 1.9%, nausea 1.3%, decreased appetite 1.3%). Due to the results reported in pancreatic cancer patients as part of the above phase I trial, the FDA has granted fast-track designation status to IBI343 as monotherapy, the first anti-CLDN18.2 ADC to receive such a designation [100].

The world eagerly awaits results from the phase III trials for AZD0901 and LM-302. If these results are promising, mG/GEJ patients with previous CLDN18.2 mAb exposure may have additional therapeutic options that target CLDN18.2 in the second-line setting and beyond. Continued success with AZD901, LM-302, and IBI343 in patients with lower CLDN18.2 expression mG/GEJ than the zolbetuximab cutoff may allow more patients access to these therapies. Potential bystander effects of ADCs may prove beneficial for tumors with heterogeneity in CLDN18.2 expression since surrounding tissues would also have localized effects from treatment. The increased potential for hematologic adverse effects of ADCs may make their use in combination with chemotherapy challenging. Additional ADCs currently under investigation for the treatment of CLDN18.2-positive G/GEJ cancer are included in Table 2 [67,68,69,70,71,72,73,74,75,76,77,78,79,80,81,82,83,84,85,86].

### 2.4. Upcoming Investigation in Anti-Claudin Agents

#### 2.4.1. Chimeric Antigen Receptor T-Cells

##### CT041 (Satricabtagene Autoleucel)

CT041, or satricabtagene autoleucel, is an anti-CLDN18.2 CAR-T product currently under investigation for the treatment of CLDN18.2-positive digestive cancers, including gastric cancer (GC) [101]. After promising results in a single-center phase I trial, a multi-center phase I trial was initiated to determine the safety and efficacy of CT041 in patients with CLDN18.2-positive advanced digestive system cancers [102]. Thirty-seven patients received a lymphodepleting (LD) regimen of fludarabine and cyclophosphamide with nab-paclitaxel or gemcitabine prior to CT041 cell infusion, and 48.6% of patients received more than one cycle of LD and CT041. From a safety standpoint, the most frequent Grade 3 or higher TRAEs reported with LD/CT041 treatment were hematologic toxicities associated with the LD regimen (100%), including leukopenia (83.8%), neutropenia (67.6%), anemia (40.5%), and thrombocytopenia (16.2%). Grade 1–2 CRS was reported in 94.6% of patients. No ICANS and no grade 3 or higher CRS was observed. One patient reportedly experienced grade 4 gastrointestinal hemorrhage due to rapid tumor regression after a second CT041 cell infusion, and one patient had anaphylactic shock after a second infusion of CT041. Preliminary efficacy results in patients with GC showed an ORR of 57.1% and a DCR of 75%. In the GC group, the median PFS was 4.2 months, and the OS rate was 81.2%. Additional trials are being conducted to confirm the promising tolerability and efficacy of CT041 in patients with GAC as well as pancreatic cancer [103,104].

Data from CAR-T trials are currently still maturing, although a myriad of compounds are currently being studied; however, CRS and neurotoxicity potential, as well as the cost of treatment, including supportive care, lymphodepletion, and inpatient stay, may limit real-world applicability in patients with G/GEJ cancers. Because CAR-T therapies require such close monitoring and potentially complex management of toxicities, access to CAR-T products for the treatment of G/GEJ cancer will likely be limited to large oncology centers. Additional anti-CLDN18.2 CAR-T therapies currently under investigation for the treatment of CLDN18.2-positive G/GEJ cancers are included in Table 3 [105,106,107,108,109,110,111,112,113,114,115,116,117,118,119,120,121].

## 3. Conclusions and Future Directions

As evident by the multitude of studies targeting CLDN18.2, CLDN18.2 positivity will be an important biomarker for the future of metastatic GAC treatment. Coupled with an incidence upwards of 40% in metastatic GAC, understanding where each CLDN18.2 targeting therapy will align is the key to success in this area. With currently overlapping trial designs with non-comparators, the future state for CLDN18.2 mAbs will likely rely on adverse effect profile, cost, ease of use, and any potential differences in binding affinity leading to better efficacy. BsAb, ADCs, and CAR-T therapy are currently in the early stages of development. Assays to determine CLDN18.2 positivity are currently unique to each agent/trial, so standardization of CLDN18.2 assays will be needed should additional agents be commercially approved. Questions needing answers to shape the future landscape of anti-CLDN18.2 therapies include: (1) Will there be different CLDN18.2 expression levels required for efficacy? (2) How will these therapies be tolerated? (3) Will there be any activity in patients previously exposed to CLDN18.2-directed therapy? (4) As seen with HER2-positive GAC, will CLDN18.2 GAC patients show inter- and intra-tumoral heterogeneity? (5) What resistance patterns are we to expect with these agents? (6) Will these therapies play a role in the treatment of GAC patients with resectable disease?

More translational understanding of the impact of these differing classes of drugs concerning the CLDN18.2 target will hold the key to answering these questions. We believe trial designs should focus on separating low, moderate, and high CLDN18.2 expression levels with either escalation in therapy (i.e., the addition of other combination strategies or utilizing more biomarker/cytotoxic drug combinations). Additionally, once progression on anti-CLDN18.2 therapy occurs, re-screening for CLDN18.2 expression may help us to understand the impact of rechallenging patients with anti-CLDN18.2 therapy in the refractory setting. We look forward to more research on this exciting and growing area of metastatic GAC treatment.

## Figures and Tables

**Table 1:** Zolbetuximab trial summaries [17,18,19,20,21].

Study Name (Trial Number)	Phase	Patient Number	Treatment Line	CLDN18.2+Cells (2+/3+ IHC)	Intervention	ORR (%)	PFS (Months)	OS (Months)
MONO (NCT04396821)	I/IIa	54	≥2nd	≥50%	ZOL	9	NR	NR
FAST (NCT01630083)	II	246	1st	≥40%	EOX +/− ZOL	25 vs. 39	5.3 vs. 7.5 HR 0.4 *p* < 0.0005	8.3 vs. 13 HR 0.55 *p* < 0.0005
ILUSTRO (NCT03505320)	II							
- Cohort 1 - Cohort 2 - Cohort 3		30	≥3rd	≥50%	ZOL	0	1.54	5.62
		21	1st	≥50%	FOLFOX + ZOL	71.4	17.81	NR
		3	≥2nd	≥50%	ZOL + PEM	0	2.96	NR
SPOTLIGHT (NCT03504397)	III	566	1st	≥75%	FOLFOX +/− ZOL	62.1 vs. 60.7	8.7 vs. 10.6 HR 0.75 *p* = 0.0066	6.8 vs. 8.2 HR 0.69 *p* = 0.0007
GLOW (NCT03653507)	III	507	1st	≥75%	CAPOX +/− ZOL	48.8 vs. 53.8	638 vs. 8.2 HR 0.69 *p* = 0.0007	12.2 vs. 14.4 HR 0.77 *p* = 0.0118

Abbreviations: CLDN = claudin; IHC = immunohistochemistry; ORR = objective response rate; DCR = disease control rate; PFS = progression-free survival; OS = overall survival; ZOL = zolbetuximab; FOLFOX = modified 5-FU/Leucovorin/Oxaliplatin; EOX = epirubicin/oxaliplatin/capecitabine; PEM = pembrolizumab; NR = not reported; CAPOX = capecitabine/oxaliplatin; vs. = versus; HR = hazard ratio.

**Table 2:** Additional Anti-CLDN18.2 Agents in Clinical Trials [67,68,69,70,71,72,73,74,75,76,77,78,79,80,81,82,83,84,85,86].

Drug Name	Trial	Type/Target	Phase	Population
AB011	NCT04400383	mAb	I	Advanced solid tumors
DR30303	NCT05639153	mAb	I	Advanced solid tumors
MIL93	NCT04671875	mAb	I	Advanced solid tumors
NBL-015	NCT05153096	mAb	I	Advanced solid tumors
SPX-101	NCT05231733	mAb	I	Advanced solid tumors
TORL-2-307-MAB	NCT05159440	mAb	I	Advanced solid tumors
ZL-1211	NCT05065710	mAb	I	Advanced solid tumors
AZD5863	NCT06005493	BsAb: CLDN18.2/CD3	I	Advanced solid tumors
SG1906	NCT05857332	BsAb: CLDN18.2/CD47	I	Advanced solid tumors
ASP2138	NCT05365581	BsAb: CLDN18.2/CD3	I	Gastric, gastroesophageal junction, and pancreatic cancer
PT886	NCT05482893	BsAb: CLDN18.2/CD47	I/II	Gastric, gastroesophageal junction, and pancreatic cancer
RC118	NCT04914117 NCT05205850 NCT06038396	ADC: Microtube INH	I I/II I/II	Advanced solid tumors
SHR-A1904	NCT04877717	ADC: Topo I INH	I	Advanced solid tumors
SKB315	NCT05367635	ADC: Topo I INH	I	Advanced solid tumors
JS107	NCT05502393	ADC: MMAE	I	Advanced solid tumors
TORL-2-307-ADC	NCT05156866	ADC: MMAE	I	Advanced solid tumors
TQB2103	NCT05867563	ADC: undisclosed	I	Advanced solid tumors
LB4330	NCT05707676	anti-CLDN18.2/CD8 T cell activator fusion protein	I	Advanced solid tumors

Abbreviations: mAb = monoclonal antibody; BsAb = bispecific antibody; ADC = antibody-drug conjugate; Topo I INH = topoisomerase I inhibitor; MMAE = monomethyl auristatin E.

**Table 3:** Anti-CLDN18.2 Chimeric Antigen Receptor T-cell Clinical Trials [105,106,107,108,109,110,111,112,113,114,115,116,117,118,119,120,121].

Drug Name	Trial	Phase	Population
AZD6422	NCT05981235	I/II	Advanced solid tumors
CT048	NCT05393986	I	Advanced solid tumors
Dual-targeting CLDN18.2/PD-L1	NCT06084286	I	Advanced solid tumors
HEC-016	NCT05277987	I	Gastric, gastroesophageal junction, and pancreatic cancer
IBI345	NCT05199519	I	Advanced solid tumors
IMC002	NCT05946226 NCT05472857	I I	Advanced digestive system tumors Advanced solid tumors
IMC008	NCT05837299	I	Advanced solid tumors
KD-496	NCT05583201 NCT06134960	I I	Advanced solid tumors Advanced solid tumors
LB1908	NCT05539430	I	Gastric, gastroesophageal junction, and pancreatic cancer
LY011	NCT04977193	I	Gastric cancer
RD07	NCT05284968	I	Advanced solid tumors
TAC01-CLDN18.2	NCT05862324	I/II	Advanced solid tumors
XKDCT086	NCT05952375	N/A	Advanced solid tumors
CLDN18.2 CAR-T (unnamed)	NCT05620732	N/A	Gastric and pancreatic cancer
CLDN18.2 CAR-T (unnamed)	NCT06353152	I	Gastric and gastroesophageal junction cancer

## Data Availability

No new data were created or analyzed in this study. Data sharing is not applicable to this article.
